# Supplementary material for: Modeling glioblastoma heterogeneity as a dynamic network of cell states
Source: Mol Syst Biol. 2021 Sep 16;17(9):e10105. doi: 10.15252/msb.202010105 (PMC8444284; doi:10.15252/msb.202010105)
Supplement: Supplementary file 6 — Source Data for Figure 5 [file MSB-17-e10105-s004.zip › Figure5A_sourcedata/GSEA_3017/nosigdb_stateB.GseaPreranked.1622795021725/NB_KI_CLUSTER7.html]

Details for gene set NB\_KI\_CLUSTER7[GSEA]

|  || Dataset | state43017 |
| Phenotype | NoPhenotypeAvailable |
| Upregulated in class | na\_neg |
| GeneSet | NB\_KI\_CLUSTER7 |
| Enrichment Score (ES) | -0.34937075 |
| Normalized Enrichment Score (NES) | -1.8734554 |
| Nominal p-value | 0.010471204 |
| FDR q-value | 0.018636476 |
| FWER p-Value | 0.078 |
Table: GSEA Results Summary

  

Fig 1: Enrichment plot: NB\_KI\_CLUSTER7      
 Profile of the Running ES Score & Positions of GeneSet Members on the Rank Ordered List

  

| PROBE | GENE SYMBOL | GENE\_TITLE | RANK IN GENE LIST | RANK METRIC SCORE | RUNNING ES | CORE ENRICHMENT || 1 | CCDC15 |  |  | 197 | 0.419 | -0.1967 | No |
| 2 | PKP4 |  |  | 311 | 0.342 | -0.2926 | Yes |
| 3 | RANGAP1 |  |  | 312 | 0.342 | -0.2358 | Yes |
| 4 | LMF2 |  |  | 338 | 0.330 | -0.2148 | Yes |
| 5 | UBR7 |  |  | 340 | 0.330 | -0.1615 | Yes |
| 6 | KMT5A |  |  | 373 | 0.318 | -0.1520 | Yes |
| 7 | TEDC1 |  |  | 399 | 0.309 | -0.1346 | Yes |
| 8 | CEP295 |  |  | 402 | 0.307 | -0.0864 | Yes |
| 9 | LIN54 |  |  | 411 | 0.303 | -0.0469 | Yes |
| 10 | SASS6 |  |  | 432 | 0.296 | -0.0248 | Yes |
| 11 | EED |  |  | 459 | 0.290 | -0.0118 | Yes |
| 12 | ING2 |  |  | 466 | 0.289 | 0.0280 | Yes |
| 13 | RNASEH2A |  |  | 478 | 0.285 | 0.0605 | Yes |
| 14 | METTL4 |  |  | 488 | 0.283 | 0.0953 | Yes |
| 15 | DONSON |  |  | 559 | 0.271 | 0.0456 | Yes |
| 16 | NUP85 |  |  | 563 | 0.270 | 0.0863 | Yes |
| 17 | CCDC18 |  |  | 586 | 0.267 | 0.1009 | Yes |
| 18 | ERI1 |  |  | 604 | 0.262 | 0.1215 | Yes |
| 19 | ZNF85 |  |  | 635 | 0.258 | 0.1237 | Yes |
| 20 | HMGCR |  |  | 683 | -0.257 | 0.1027 | Yes |
Table: GSEA details [plain text format]

  

Fig 2: NB\_KI\_CLUSTER7: Random ES distribution      
 Gene set null distribution of ES for **NB\_KI\_CLUSTER7**
